# Merkel cell carcinoma: updates in tumor biology, emerging therapies, and preclinical models

**DOI:** 10.3389/fonc.2024.1413793

**Published:** 2024-07-29

**Authors:** Elisabeth A. Pedersen, Monique E. Verhaegen, Mallory K. Joseph, Kelly L. Harms, Paul W. Harms

**Affiliations:** ^1^ Department of Dermatology, University of Michigan, Ann Arbor, MI, United States; ^2^ Department of Pathology, University of Michigan, Ann Arbor, MI, United States

**Keywords:** neuroendocrine carcinoma (NEC), immunotherapy, mouse model, Merkel cell carcinoma (MCC), epigenetics, viral tumorigenesis, UV signature, clinical trial

## Abstract

Merkel cell carcinoma (MCC) is an aggressive cutaneous neuroendocrine carcinoma thought to arise via either viral (Merkel cell polyomavirus) or ultraviolet-associated pathways. Surgery and radiotherapy have historically been mainstays of management, and immunotherapy has improved outcomes for advanced disease. However, there remains a lack of effective therapy for those patients who fail to respond to these established approaches, underscoring a critical need to better understand MCC biology for more effective prognosis and treatment. Here, we review the fundamental aspects of MCC biology and the recent advances which have had profound impact on management. The first genetically-engineered mouse models for MCC tumorigenesis provide opportunities to understand the potential MCC cell of origin and may prove useful for preclinical investigation of novel therapeutics. The MCC cell of origin debate has also been advanced by recent observations of MCC arising in association with a clonally related hair follicle tumor or squamous cell carcinoma *in situ*. These studies also suggested a role for epigenetics in the origin of MCC, highlighting a potential utility for this therapeutic avenue in MCC. These and other therapeutic targets form the basis for a wealth of ongoing clinical trials to improve MCC management. Here, we review these recent advances in the context of the existing literature and implications for future investigations.

## 1 Introduction

Merkel cell carcinoma (MCC) is a poorly differentiated primary cutaneous neuroendocrine carcinoma. It is among the most aggressive cutaneous solid tumors, with significant rates of disease-associated morbidity and mortality. MCC is now known to consist of two molecular subclasses: Merkel cell polyomavirus-associated and UV-associated, which are denoted as virus-positive MCC (VP-MCC) and virus-negative MCC (VN-MCC) throughout this review. Recently, immunotherapy has dramatically improved prognosis in advanced disease, but is not universally effective. Thus, there is continued need for better molecular understanding, prognostication, and therapeutic options for MCC.

In this review, we discuss the current state of MCC treatment and how it relates to the cellular and molecular pathogenesis of MCC. We also review the emerging therapeutic strategies and the related need for improved preclinical models of MCC.

## 2 Clinical management of MCC

### 2.1 Clinical presentation and diagnosis

MCC arises most frequently in fair-skinned patients, with 89.9% of MCC cases identified in White, 5.7% in Hispanic, 2.3% in Asian American or Pacific Islander, and 1.5% in Black patients (1). The greatest incidence of MCC has been noted in Australia (1.6 cases per 100,000 individuals) (2), and is in line with the observed greater MCC incidence with geographic proximity to the equator (3). In 2013, the incidence in the United States was approximately 0.7 per 100,000, which was a 95% increase since 2000 (4). MCC is more common in males compared to females (3:2, respectively) (4–6), and the median age of diagnosis is >75 years, with very rare incidence in patients < 40 years (5). For MCC, the incidence continues to rise with each additional decade of life, whereas in most other malignancies, the incidence peaks at a mid-/later decade of life and then declines with further age (2). The incidence of MCC is greater in patients with impaired immune functioning such as organ transplantation, hematologic malignancy, immunosuppressive medications, and other causes of decreased immunity (3).

MCC most often arises as a red-to-violaceous papule or nodule on sun-exposed skin. It may also arise as a subcutaneous nodule with no overlying skin changes. Tumors often grow rapidly over the course of 2-3 months. MCC has a high propensity for metastasis; up to 35% of patients will have either regional lymph node or distant metastases (7) at the time of diagnosis. Frequent sites of metastatic involvement are adjacent skin (satellite or in-transit metastases); regional lymph nodes; and distant sites including bone, visceral organs (liver, lungs), distant skin, and distant lymph nodes.

**Figure 1:**
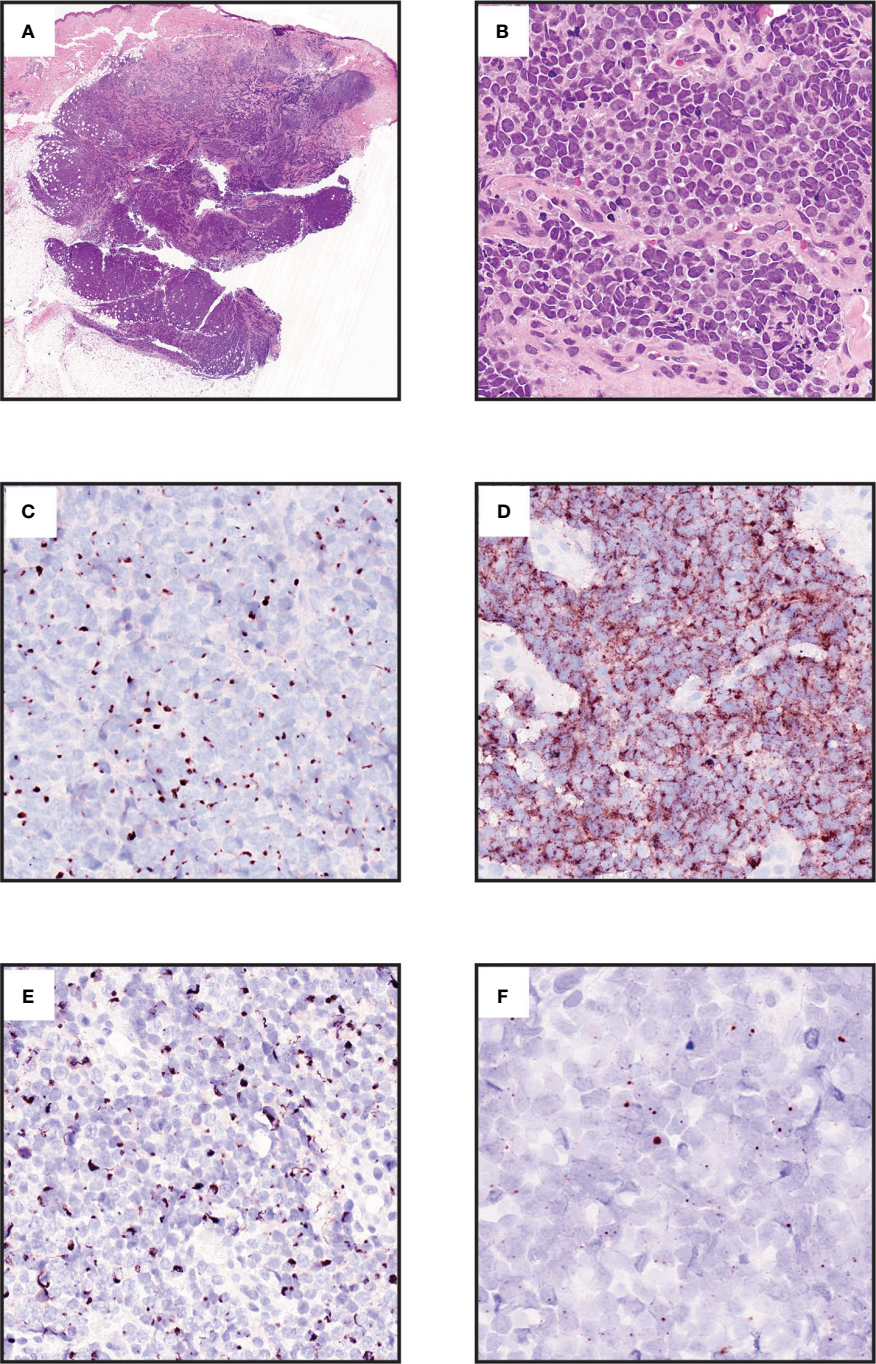
Morphologic and immunophenotypic features of Merkel cell carcinoma. **(A)** Expansile blue cell tumor occupying dermis with extension into subcutis (hematoxylin and eosin [H&E], 0.7x. **(B)** Small round cell tumor with neuroendocrine chromatin, mitotic activity, and crush artifact (H&E, 40x). **(C)** Cytokeratin 20 displays distinctive paranuclear dot labelling. (Chromogen DAB, counterstain hematoxylin, 40x). **(D)** Neuroendocrine marker Chromogranin A is immunohistochemically expressed in most cases (Chromogen DAB, counterstain hematoxylin, 40x). **(E )** Neurofilament immunohistochemistry displays paranuclear dot labelling and provides a more specific marker than cytokeratin 20 for MCC (Chromogen DAB, counterstain hematoxylin, 40x). **(F)** RNA *in situ* hybridization for the MCPyV T-antigen transcript in a virus-positive MCC (Chromogen DAB, counterstain hematoxylin, 80x).

MCC is a poorly-differentiated neuroendocrine carcinoma with round cell morphology (**Figure 1**) (2, 7–9). In most cases, the tumor is centered in the dermis, possibly with extension into the subcutis (**Figure 1A**) (7). In a minority of cases, there is pagetoid scatter in the overlying epidermis. Purely intraepidermal cases are exceptionally rare. There can be squamous metaplasia, eccrine differentiation, or rarely sarcomatoid change. A minority of VN-MCC is accompanied by SCCIS in the overlying epidermis, or less frequently invasive SCC (7, 10). Rarely, VP-MCC can be associated with other tumor types including trichoblastoma or poroma (11, 12).

MCC, like other poorly-differentiated tumors, can be challenging to distinguish from histopathologic mimics. The differential diagnosis for MCC depends on site of presentation and tumor morphology (7, 9). Extracutaneous small cell carcinoma metastatic to the skin is morphologically identical to MCC. Other small round cell tumors, such as lymphoma, small cell melanoma, or Ewing sarcoma, might also enter the differential diagnosis (7, 9). For MCC with more prominent pleomorphism, other poorly differentiated cutaneous carcinomas might also be considered (7).

Several other neuroendocrine tumors arise in the skin or subcutis and must be distinguished from MCC. Endocrine mucin-producing sweat gland carcinoma, and the related neuroendocrine subtype of mucinous carcinoma, are adenocarcinomas of the eyelid that may resemble MCC but lack round cell morphology (13). Primary cutaneous neuroendocrine tumors (carcinoids) display morphologic features of neuroendocrine tumors but are histologically lower grade than MCC (14). Recently, another category of primary cutaneous neuroendocrine carcinoma, termed sweat gland carcinoma with neuroendocrine differentiation (SCAND), has been proposed; however, these tumors also lack the round cell morphology of MCC (15).

Immunohistochemistry is critical for confirming the diagnosis of MCC and excluding histopathologic mimics (7–9). It should be noted that no single marker is completely sensitive for MCC, thus diagnosis often requires multiple immunohistochemical stains, expert evaluation, and clinical correlation to confirm the diagnosis. Commonly used markers include CK20 expression in a paranuclear dot pattern (**Figure 1C**), which is distinctive for MCC, but may not always be present (7). Neuroendocrine markers such as chromogranin A or synaptophysin are typically expressed (**Figure 1D**). Paranuclear dot labeling for neurofilament is also highly characteristic (**Figure 1E**) (7, 16, 17). Thyroid transcription factor 1 (TTF-1) and cytokeratin 7 are typically negative. Detection of MCPyV by immunohistochemistry or RNA *in-situ* hybridization supports a diagnosis of MCC (**Figure 1F**) (7, 10, 18). INSM1 is a highly sensitive marker for MCC, although specificity is limited relative to other neuroendocrine carcinomas (19–21). SATB2 has more limited sensitivity, but provides added specificity in that strong diffuse expression favors MCC over metastatic small cell carcinoma (22–24). SOX2 expression is highly sensitive for MCC (92%), and is highly specific for MCC compared to other cutaneous carcinomas, as well as 70% specific for MCC compared to other neuroendocrine carcinomas (25, 26). ATOH1 is expressed in MCC, however there is mixed data regarding the specificity of this marker for excluding other non-MCC neuroendocrine carcinomas (23, 27–30). VN-MCC has a higher rate of immunophenotypic aberrancy such as CK20 negativity (7, 31–33).

Morphology alone is usually sufficient to distinguish MCC from malignancies other than small cell carcinomas. However, several pitfalls merit mention. Neuroendocrine marker expression can be seen in some cases of other cutaneous carcinoma including basal cell carcinoma. Brisk obscuring inflammation, and/or expression of certain lymphoid markers (TdT, PAX5, SOX11, immunoglobulins) in a subset of MCC (7, 34, 35), can raise confusion with a lymphoid process. S100 expression has been described for MCC (7), and conversely can be lacking in small cell melanoma (36), potentially raising diagnostic difficulty. However, nuclear SOX10 expression is exceedingly rare in MCC, and other melanocytic markers will be negative, thus allowing reliable distinction from small cell melanoma (7, 37–40). MCC can express CD99 similar to small round cell sarcomas such as Ewing sarcoma, but will lack *EWSR1* rearrangements (2, 7, 41).

In challenging cases such as neuroendocrine carcinomas presenting in the parotid, or carcinomas of unknown primary, analyses demonstrating MCPyV or UV-signature mutations can also be useful for distinguishing MCC from extracutaneous neuroendocrine carcinomas (2, 7, 23, 42–45). Of note, the potential for primary parotid neuroendocrine carcinomas to be MCPyV-positive has been debated (44, 46–48), and thus currently clinicopathologic correlation remains essential for classifying MCPyV-positive neuroendocrine carcinomas in the parotid.

### 2.2 Prognosis

The AJCC 8^th^ edition defines staging guidelines for MCC based upon tumor size and the presence/pattern of metastatic spread (5). In addition, it is well established that advanced age, male sex, immunosuppression, and patients with a known primary lesion with clinically evident nodal metastases are associated with a higher risk of recurrence (49, 50). Additional prognostic factors are a subject of ongoing research. Many potential biomarkers for predicting MCC outcome have been proposed (2, 7, 51). Although data from smaller studies is mixed, several large studies have found that the viral status of MCC is prognostic: specifically, the presence of MCPyV is a favorable prognostic finding, with improved disease-specific and progression-free survival in VP-MCC compared to VN-MCC (2, 7, 10, 52–54). Tumor expression of p63 has been proposed to be predictive of outcomes (7, 55–58), however the clinical utility of this marker has been questioned (58). Many studies have found that immune infiltration patterns or marker expression can be informative of outcome for MCC tumors (2, 7, 10, 59, 60), especially CD8+ T-cells (7, 61–63). The predictive value of immune markers might vary based upon tumor MCPyV status (10), which was often not considered in such studies. Notably, the great majority of these studies were based upon outcomes data collected prior to the approval of immunotherapy for MCC, and thus reappraisal of these findings in the immunotherapy era will be essential.

### 2.3 Treatment

Clinical trials and the results of single- and multi-institution cohort studies provide the best evidence to support current practices for MCC treatment. Given the rarity of MCC, high-powered randomized controlled trials to identify optimal treatment approaches have not been feasible. The National Comprehensive Cancer Network (NCCN) Guidelines for MCC provides an annually updated evidence-based, and consensus-driven algorithmic approach for management of all stages of MCC (64). In addition, the European consensus-based interdisciplinary guideline was published in 2022 (65). Importantly, for patients with any stage of MCC, a multidisciplinary team is recommended as management requires the involvement of multiple specialties. Please refer to the NCCN and European Consensus guidelines for the most comprehensive and up-to-date algorithms for management (64, 65).

Initial evaluation and work-up of a patient newly diagnosed with MCC includes a thorough history with special attention directed towards immunosuppression. Immunosuppression is a known adverse prognostic factor, and it is recommended that immunosuppressive regimens are reduced by the prescribing physician when medically feasible. The initial physical exam should focus on identifying clinical staging parameters including the clinical size of the primary tumor, clinical in-transit metastases, and clinically detectable regional lymph node metastases. For patients without clinical lymph node metastases, baseline imaging with PET-CT is often recommended, as it has been shown to find additional asymptomatic sites of disease and impact staging (66). Suspected metastases should be confirmed by biopsy. Subsequent management is based upon stage of disease (64).

Surgical treatment with or without radiotherapy is the current standard of care. Due to the lack of randomized controlled trials, the optimal surgical margin is not well defined. The current NCCN recommendation is for excision with 1-2 cm margins, with Mohs micrographic excision (excision done by stages with frozen section evaluation of margins) designated as useful in certain circumstances (64). The risk of occult metastasis is high in patients presenting without clinically detected regional lymph node metastasis, and sentinel lymph node biopsy (SLNB) is recommended at the time of surgical removal of the primary tumor. Studies have demonstrated SLNB positivity rates as high as 26 - 45% (67–69) in institution based-cohorts. Observation is recommended for patients with a negative SLNB result, with evidence supporting a low risk of regional recurrence after a negative SLNB (70). For management of SLNB-positive disease, current guidelines recommend further treatment including lymph node dissection or radiation therapy (64), which have both demonstrated association with low rates of regional recurrence (71, 72).

MCC is highly sensitive to radiation therapy, and adjuvant radiation is recommended in cases of positive surgical margins (versus re-excision) and in the presence of adverse risk factors, such as primary tumors > 1 cm in size, immunosuppression, head/neck site, or lymphovascular invasion. For patients who are not surgical candidates, the recommendation is for multidisciplinary discussion and consideration of definitive radiation therapy or systemic therapy (64).

For patients with clinically detected lymph node metastases prior to surgery, a lymph node biopsy via fine needle aspiration or core needle biopsy is recommended to confirm the presence of metastatic MCC. For treatment of biopsy-confirmed clinically detectable lymph node metastases, current NCCN guidelines recommend clinical trial, or combination of lymph node dissection and radiation therapy. In addition, neoadjuvant therapy (e.g. treatment prior to surgery) with immunotherapy is emerging as a potential option for treatment.

Reports of immune-mediated spontaneous regression in some cases of MCC suggested the potential for therapeutic harnessing of antitumor immunity for advanced disease (2). Immunotherapy, also known as immune checkpoint inhibitor therapy, has become widely used in management of metastatic MCC and preferred over traditional chemotherapy due to improved responses and more favorable side effect profiles. Three immune checkpoint inhibitors are currently FDA-approved for the management of metastatic or advanced MCC. Avelumab, a monoclonal antibody targeted against programmed cell death ligand 1 (anti-PD-L1) was FDA-approved in 2017 for metastatic MCC. The JAVELIN Merkel 200 trial part A showed a 32% objective response rate to second-line avelumab (73); long-term follow up demonstrated a median overall survival (OS) of 12.6 months and a 5-year OS of 26% (74). Results of JAVELIN Merkel 200 part B showed a 62% objective response rate (ORR) to first-line avelumab (75). More recently, a retrospective review of patients with advanced MCC initiated on avelumab reported durable response rates as high as 73% (76). Pembrolizumab and retifanlimab, monoclonal antibody therapies targeting programmed cell death protein -1 (PD-1), were FDA-approved in 2018 and 2023, respectively, for recurrent, locally advanced and metastatic MCC (77). In the Keynote-017 trial, pembrolizumab demonstrated 56% overall response rate (ORR) (78); long-term follow-up demonstrated a 24-month overall survival rate of 68.7% (79). The PODIUM-201 trial demonstrated a 46.2% ORR, and 62% of patients had a response of ≥ 12 months (80). Nivolumab, an anti-PD-1 antibody, is also showing promise in the treatment of MCC, with numerous ongoing studies evaluating the efficacy of single and multi-agent therapy (2, 81).

Emerging neoadjuvant studies have shown promising results and are being recommended more frequently. The Checkmate 358 trial demonstrated that half of patients with clinically detectable lymph node metastases who received neoadjuvant nivolumab responded with tumor reductions of > 30% (82). NCCN guidelines currently recommend multidisciplinary discussion for consideration of neoadjuvant immunotherapy (64).

Overall, evidence across studies suggests that immunotherapy can achieve durable response in a significant proportion of patients with advanced MCC, especially when used as first-line therapy (83). However, patient factors including advanced age often seen in MCC patients and morbidity from treatment may alter treatment course or deem immunotherapy a less favorable option. Additional investigations with longer follow-up times in larger cohorts will be essential for more definitive assessment of durable response rates across different patient groups.

Management of metastatic MCC has historically included chemotherapy with etoposide and carboplatin/cisplatin-based regimens; however, the response to chemotherapy is not durable and most patients experience relapse. In a retrospective study of patients with metastatic MCC, first-line chemotherapy had a response rate was 55%, but also had a median progression free survival of 94 days (84). For second-line chemotherapy, the response rate was 23% with a median progression free survival of 61 days. Given initial response rates, chemotherapy may be useful in certain circumstances, such as for those who cannot undergo immune checkpoint inhibition due to immunocompromised status or the presence of a comorbid autoimmune condition (2, 85). It may also serve as a palliative therapy to slow the rate of rapidly growing symptomatic tumors or metastases.

As immunotherapy is not universally effective in MCC, predicting treatment response could be helpful to avoid the risk of immunotherapy toxicity in patients who will not benefit from therapy, and to consider alternative treatments if those become available. However, predictors of MCC response to immunotherapy have been elusive. Responses have not correlated with tumor PD-L1 expression (73, 78, 82, 86, 87), MCPyV status (73, 82, 86–89), tumor mutational burden (82, 86, 87), or UV mutation signatures (87). A retrospective study showed that single nucleotide variants of *ARID2* and *NTRK1* were associated with a better response to immunotherapy (87). More sophisticated approaches may be informative, such as characterizing immune populations (especially T-cells) (88, 90–95) or measuring spatial associations of cell populations in the tumor microenvironment (88, 96). However, to date none of these approaches have reached routine clinical use for decisions on immunotherapy eligibility.

After definitive treatment with surgery, radiation, and/or systemic therapy, close surveillance is typically recommended. Due to the significant risk of recurrence, current management for MCC includes imaging (such as by CT) every 3-6 months for several years following diagnosis (64). Alternative and emerging methods for disease surveillance include circulating burden of antibodies against MCPyV T-antigens (64, 77, 97, 98), and circulating tumor DNA (77, 99).

## 3 Molecular and cellular pathogenesis of MCC

### 3.1 Overview of molecular alterations in MCC

The above average prevalence of MCC in immune-compromised transplant and HIV patients (100) alluded to a possible viral etiology. This possibility was confirmed when analysis of MCC tumors via digital transcriptome subtraction methodology identified viral transcripts from a previously unknown human polyomavirus, which the authors thus named the Merkel cell polyomavirus (MCPyV) (101). The initial finding that only about 80% of tumors had integrated viral sequences suggested that a fraction of MCCs arise by a distinct mechanism. Following additional studies, two often histologically indistinguishable molecular subclasses have now been defined: MCPyV associated (virus-positive MCC; VP) and UV-associated (virus-negative MCC; VN) tumors (2, 43, 45, 102).

### 3.2 The Merkel cell polyomavirus and viral-associated MCC

The MCPyV belongs to the *Polyomaviridae* (PyV) family of small non-enveloped, circular, double-stranded DNA viruses, of which to date, 15 have been isolated from human samples with 13 identified as genuine human viruses (103). Several polyomaviruses are associated with cutaneous infections (trichodysplasia spinulosa polyomavirus, human polyomavirus 6, and human polyomavirus 7) and other human diseases (JC Polyomavirus, BK polyomavirus). However, MCPyV is the sole family member classified as a Group 2A carcinogen (probably carcinogenic to humans) by the International Agency for Research on Cancer (104). Polyomaviruses are widely prevalent in the human population, and MCPyV can be detected in the skin of most healthy individuals and at low levels in a wide variety of tissues. Serological studies indicate primary infection occurs most likely in early childhood and seroprevalence in adults is typically 60–80% (105).

During the MCPyV infectious cycle, the MCPyV helicase (C-terminal domain of LTAg) mediates replication of viral DNA by recruiting the host cell machinery. The viral DNA of MCPyV can be repeatedly and rapidly amplified during a single cell cycle, referred to as unlicensed replication (106). The host cell factors that contribute to MCPyV replication are poorly understood. In healthy populations, antiviral responses induced by MCPyV, such as IFN production, may inhibit early transcription and restrict propagation, whereas skin injury or UV irradiation may induce growth factors promoting viral propagation, thus providing a way to support persistent MCPyV infection (107, 108). Degradation of LTAg by ubiquitin ligases, which may be abrogated by cellular stresses, is another proposed mechanism for latency and reactivation of MCPyV (109–112), although this mechanism has been debated (113).

Despite a lifelong history of MCPyV exposure and infection in most individuals, development of MCC tumors remains remarkably rare. Productive viral infection is associated with host cell death (114), precluding the possibility of cell transformation during the normal viral life cycle. Two critical events are thus hypothesized to precede MCPyV-driven transformation: accidental integration of the viral genome into the host genome, and loss of viral replicative capacity due to LTAg mutations which yield a non-replicative truncated LTAg (tLTAg) (101, 115). The clonal pattern of viral integration suggests infection and integration occurs before tumor expansion, providing evidence for MCPyV being causal for tumorigenesis (101).

### 3.3 MCPyV T antigens

Like other polyomaviruses, the MCPyV genome consists of an early region (ER) coding for regulatory proteins involved in replication and transcription, a late region (LR) coding for structural proteins, and a non-coding control region (NCCR) containing the origin of DNA replication, regulatory elements, and transcription promoters (116). Four spliced transcripts are produced by the ER that code for four proteins: the large T antigen (LTAg) and the alternative splicing product 57kT; the small T antigen (sTAg); and alternate frame of the LTAg open reading frame (ALTO, as analogous to middle T antigen or MT)(**Table 1**) (101, 129, 131). The LR encodes two capsid proteins (VP1 and VP2) required for viral assembly, and a microRNA targeting the TAg transcripts (132–134).

**Table 1:** MCPyV T antigen gene products, domains, and functions.

MCPyV T antigens	Key structural components	Associations and Functions
**sTAg (ST)** Small T Antigen	N-terminal DnaJ domain LTAg stabilization domain (LSD) PP2A binding domain	LSD is required for oncogenesis *in vitro* and *in vivo*. Regulates protein stability of oncoproteins including LTAg (109, 117–119). LSD domain inhibits E3 ubiquitin ligases (109). Acts in complex with MYCL and EP400 (120). The role of the PP2A binding domain is currently unclear and possibly dispensable (117, 118, 121).
**LTAg (LT)** Large T antigen	N-terminal DnaJ domain MCPyV-unique region (MUR): *LXCXE retinoblastoma-associated protein (RB1) binding domain* *Nuclear localization signal (NLS)* *Origin binding domain (OBD)* *C-terminal helicase/ATPase domain*	LXCXE binding motif drive proliferation via inactivation of RB and subsequent activation of E2F cell cycle genes (2, 115, 122). Forced LTAg expression promotes reprogramming toward a Merkel cell-like lineage via induction of ATOH1 and SOX2 (123–125). LTAg expression is dependent upon and regulates neuroendocrine phenotypes (126).
**tLTAg (tLT)** Truncated Large T antigen	In MCPyV integrated within MCC cells, the LTAg gene is affected by a truncating mutation or deletion. *DnaJ domain and the LXCXE binding motif are retained* *OBD and helicase domain are eliminated* The exact mutation or deletion event resulting in truncation is highly variable among individual tumors.	LXCXE (Rb binding domain) contributes to virus-driven cellular proliferation (2, 122). Deletion of the OBD and helicase domain render the MCPyV non-replicative (2, 115, 127, 128).
**ALTO** Alternate frame of the LTAg open reading frame	Translated from an internal start site in the second exon of LTAg Possesses a distinct C-terminus Similarity to middle T antigen	ALTO likely has structural and functional similarities to the murine polyomavirus MT (129). May target Src family kinases, leading to activation of the NF-κB inflammatory signaling pathway, possibly impacting viral replication (130).

Evidence suggests that tLTAg and sTAg both contribute to the oncogenic potential of MCPyV (117, 118, 135–140). Expression of these oncoproteins is required for VP-MCC cell growth and survival (141, 142). Interestingly, while LTAgs from most other polyomaviruses such as SV40 typically demonstrate transforming ability (143), the MCPyV tLTAg is not sufficient to drive transformation alone *in vitro (* 135) or *in vivo* (118), but rather cooperates with sTAg to drive transformation (137).

The LTAg contains several functional domains, most notably the LXCXE retinoblastoma-associated protein (RB1) binding domain, and C-terminal region responsible for mediating viral replication [reviewed in (144)]. In MCPyV integrated within MCC cells, the LTAg gene is affected by a truncating mutation or deletion to generate tLTAg, eliminating the C-terminus and rendering the virus non-replicative (101, 115, 145) while sparing the LXCXE binding motif (2, 146), which plays a critical role in LTAg-driven proliferation via inactivation of RB and subsequent activation of E2F cell cycle genes (2).

In contrast to sTAgs of other polyomaviruses, the MCPyV sTAg displays transformation capability in culture (135) and *in vivo* (117, 118, 139). Several potential mechanisms for this activity have been investigated, including promoting activity of the mTOR pathway (135), regulation of protein stability via a unique LTAg stabilization domain (LSD) of sTAg proposed to inhibit E3 ubiquitin ligases and stabilize oncoproteins including LTAg (109, 117–119), cell surface localization of proteins involved in tumor cell recognition and tumor microenvironment regulation such as CD47 (147), and interacting with protein phosphatases and the NF-kB essential modulator (NEMO) to achieve inhibition of NF-kB signaling, a potential mechanism for repressing inflammatory signaling (148).

One of the most recently described roles for sTAg may have an even more profound influence on cellular functions. MCPyV sTAg can complex with the MYC homolog, L-MYC and its binding partner MAX, which in turn recruit the chromatin remodeling complex EP400 (120). The sTAg-MYCL-EP400 complex transcriptionally activates hundreds of downstream targets involved in ribosomal biogenesis, splicing, glycolysis, and other metabolic functions. This complex also increases levels of the mouse double minute 2 homolog (*MDM2*) E3 ubiquitin-protein ligase, which binds p53 and promotes its ubiquitination and proteasomal degradation, as well as CK1α, an activator of MDM4 (120, 149). Thus, although VP-MCC typically harbor wild-type *TP53* (43), p53 is indirectly inactivated by MCPyV sTAg via this mechanism. Additional activated targets of the sTAg-MYCL-EP400 complex include LSD1, RCOR2 and INSM1, which form part of a transcriptional repressor complex that opposes the ncBAF complex involved in differentiation, and the ATOH1 transcription factor which governs Merkel cell fate (150). The sTAg complex has also been shown to upregulate genes involved in cell motility, and downregulate genes involved with cell adhesion properties, revealing possible sTAg roles in invasion and metastasis (120, 140, 151–153).

Analogous to other sTAgs, the MCPyV sTAg contains a PP2A binding domain. However, the role of the PP2A binding domain in sTAg-mediated transformation is currently unclear and possibly dispensable (117, 118, 121).

To date, the functional role of MCPyV ALTO has not been well recognized, but emerging evidence now suggests that ALTO has structural and functional similarities to the murine polyomavirus MT, targeting Src family kinases, leading to activation of the NF- κB inflammatory signaling pathway, and possibly impacting viral replication (130).

While not yet functionally understood, significant levels of intrinsic protein structure disorder have recently been described in sTAg, LTAg, ALTO, 57kT, and VP1, characterized by the lack of a consistent three-dimensional structure (154). These disordered structures may present MCPyV viral proteins with unique functional properties including exceptional binding promiscuity and interactions with unexpected partners (154). This feature might contribute to the high diversity of functions that have been attributed to MCPyV T antigens.

### 3.4 UV-associated virus negative MCC

Initial focused sequencing studies on MCC identified recurrent mutations including *TP53* and *RB1* in a subset of tumors but did not correlate these findings with MCPyV or global mutation changes (54, 155–159). However, later studies that broadly examined the MCC mutational landscape in the context of MCPyV immediately identified two molecular subclasses with significant differences in mutational burden (43, 45, 102). Virus-negative tumors displayed a high UV-signature mutational burden with recurrent inactivation of tumor suppressor genes *TP53* and *RB1*, while virus-positive tumors had an extremely low mutational burden (43, 45, 102). VN-MCC can present with a mutational burden of 40 mutations per megabase, which is higher than any cancer sequenced by The Cancer Genome Atlas and more than 100-fold higher than VP-MCC (43, 102). Metastatic VN-MCC tumors exhibit additional genomic complexity (160). In contrast, VP-MCC typically have relatively normal diploid genomes with few somatic mutations (43, 45, 102), and those present are often subclonal, suggesting they occurred after tumor initiating events (161). Mutations in UV-associated VN-MCC often result in inactivation of genes involved in several signaling pathways, including NOTCH (*NOTCH1, NOTCH2*), DNA damage repair (*KMT2A*, *KMT2C KMT2D*, *ASXL1*, *ARID1A*, *ARID1B*, *SMARCA4)* and chromatin-modifying pathways (*ATM*, *MSH2*, *BRCA1*, *BRCA2*, and *BCOR) (* 10, 43, 45, 86, 102, 146 *).*

Oncogene activation events tend to be detected with greater frequency in VN-MCC than VP-MCC and are overall heterogeneous (10, 86). The most highly recurrent oncogene activation events include *MYCL* amplification and *PIK3CA* activating point mutation. Other hotspot activating mutations reported in MCC include *TERT* promoter, *KNSTRN*, *RAC1*, *HRAS, KRAS, NRAS*, *AKT1*, *CTNNB1, IDH1, IDH2* and *EZH2*, among others (2, 42, 43, 45, 86, 102, 157, 162, 163).

At the transcriptional level, VN-MCC shows distinct patterns of gene expression from VP-MCC (10, 164–168), including upregulation of genes involved in the epithelial-to-mesenchymal transition and developmental processes, and downregulation of genes involved in neuroendocrine and epidermal differentiation, which may contribute to its more aggressive nature (165, 167).

### 3.5 Epigenetic changes in MCC

Epigenetic modifications encompass a spectrum of reversible covalent modifications, especially to histone proteins and DNA, that exert powerful effects on chromatin accessibility and hence gene expression (169, 170). Alterations in epigenetic patterning can contribute to the hallmarks of cancer, and are frequently observed in malignancies (169–171).

A consistent model for the contribution of epigenetic dysregulation to MCC tumor biology has remained elusive. Although many sequencing studies have identified mutations involving chromatin modifier genes in MCC, the genes involved are heterogeneous with no highly recurrent mutational driver of epigenetic dysregulation (10, 42, 43, 45, 86, 102, 161, 172, 173). Until recently there have been relatively few studies establishing patterns and significance of epigenetic alterations in MCC. However, recent studies have provided new insights into the biological importance of the tumor epigenome in MCC.

Methylation of DNA occurs on cytosine residues and is a major regulator of gene expression. Methylation of promoter CpG islands and adjacent shores is associated with silencing, whereas methylation of gene body sites is associated with increased expression (174–176). Patterns of DNA methylation can yield insights into tumor classification and cell of origin (177). DNA methylation is perturbed in tumorigenesis, including global methylation changes and specific silencing of tumor suppressor genes (171). DNA hypomethylation agents have been approved as antitumor therapy for some myeloid neoplasms and are under investigation as therapeutics for solid tumors (178, 179), underscoring the mechanistic importance of altered DNA methylation in tumor biology.

Until recently, studies into epigenetic patterning in MCC have been limited to specific genes. Promoter hypermethylation has been described for tumor suppressor genes including *CDKN2A, RASSF1, RB1, DUSP2*, and *MGMT* (180–184), although protein expression studies have not shown that these genes are consistently silenced in MCC tumors (53, 164, 182, 184, 185). Hypomethylation of the *PTCH1* tumor suppressor has been described in MCC, but this did not consistently correlate with gene expression (186). Thus, the biological significance of these methylation events has remained unclear. The cell surface protein PD-1 (encoded by *PDCD1*), long known to be associated with immune exhaustion when expressed on T-cells, has recently been proposed to also be a tumor suppressor expressed on some tumor cells (187). *PDCD1* promoter hypermethylation has been described in MCC, in which context it may correlate with reduced immune response and worse prognosis (188).

Recent studies have provided greater detail on patterns of DNA methylation in MCC (189–192). Based on global genomic patterns, MCC assorts into a distinct cluster from other tumor types, with closest similarity to small cell lung carcinoma, and relative similarity to squamous cell carcinoma (189). Comparison to other tumor types by a score-based approach also demonstrated the epithelial and neuroendocrine-type patterning of DNA methylation in MCC (190). VP-MCC displays distinctive genome-wide DNA methylation patterns as compared to VN-MCC, although the magnitude and significance of such differences is unclear (189–191). In particular, VP-MCC displays globally decreased methylation compared to VN-MCC, as indicated by direct measurement of genomic methylation, and by expression of the hypomethylation marker long interspersed nuclear element-1 (189, 193). The mechanism for DNA methylation dysregulation in MCC remains unclear, as DNA methyltransferase genes (DNMTs) are not consistently overexpressed or mutated in MCC (43, 45, 102, 189). Nonetheless, promoter methylation might provide a mechanistic explanation for expression patterns of many diagnostic and prognostic markers in MCC (189, 191). There is also evidence that DNA methylation patterns change with MCC progression (191). DNA methylation may also coordinate or promote other epigenetic changes in MCC such as histone methylation and acetylation (189, 191). In support of a biological role for DNA methylation in MCC biology, treatment with the DNA hypomethylating agent decitabine is associated with antiproliferative effects in VN-MCC and cell death in VP-MCC (189).

In addition to this proposed role in cell proliferation and survival, DNA methylation also appears to be a mechanism of immune evasion for MCC (189). HLA presentation of “non-self” antigens, either viral antigens for VP-MCC or UV-mutational neoantigens for VN-MCC (2), represents a potentially powerful trigger for antitumor immunity against MCC. HLA presentation can be downregulated by direct silencing of HLA genes and/or by repressing expression of the antigen presenting machinery responsible for mediating cell-surface localization and antigen presentation by HLA. In MCC, promoter hypermethylation is observed for HLA and antigen presenting machinery genes (189). This is likely functionally significant, as treatment by a hypomethylating agent resulted in de-repression and restored membrane localization of HLA (189). Therefore, hypomethylating agents might increase the antigenicity of MCC to promote antitumor immunity.

The reversal of DNA methylation is achieved via an intermediate step in which TET enzymes convert 5-methylcytosine to 5-hydroxymethylcytosine (5-hmc). The dysregulation of TET activity plays a significant role in driving some hematopoietic neoplasms (194). Inactivating mutations of *TET2*, and activating mutations of *IDH1/IDH2* that inhibit TET activity, can occur in MCC but are uncommon relative to other potential drivers (10, 42, 86). Global loss of 5-hmc has been reported in aggressive cases of MCC (195), although the mechanism for TET dysregulation in such cases remains unclear.

Histone modifications, especially methylation, phosphorylation, and acetylation, can play roles in either promoting or silencing gene expression (170, 196). Histone acetylation on lysine residues promotes chromatin relaxation to facilitate gene expression, and is regulated by histone acetyltransferases (HATs) and histone deacetylases (HDACs). Histone methylation (either mono-, di-, or tri-methylation) on lysine and arginine residues represents a complex multistep regulatory process that generally promotes closed chromatin and transcriptional repression (170). However, some histone methylation marks such as H3K4 trimethylation are associated with active transcription. Histone methylation is regulated by histone methyltransferases (including SET domain proteins such as the MLL family) and demethylases such as LSD1. Analogous to DNA methylation, histone modification can be a mechanism for tumor suppressor silencing or oncogene overexpression, and thus histone modifiers such as histone deacetylases represent therapeutic targets in tumors (170).

The Polycomb repressor complex 2 (PRC2) silences genes via repressive trimethylation of Histone 3 lysine 27 (H3K27Me3) (197). Dysregulated activity of PRC2, especially the catalytic subunit EZH2, has been implicated in many malignancies (197). EZH2 inhibitors display antitumor activity in epithelioid sarcoma and follicular lymphoma (198, 199). The role of PRC2 activity in MCC may be more complex. During embryogenesis, PRC2 acts to oppose normal Merkel cell development via silencing of the master Merkel cell fate regulator SOX2 (200). Global H3K27Me3 loss can be observed in many MCC consistent with loss of PRC2 activity (173, 201–204). H3K27Me3 loss occurs in tandem with the transition from a squamous progenitor population into MCC in at least some cases (173), suggesting similar roles for PRC2 in antagonizing both normal Merkel cell development and the emergence of MCC.

Given this evidence that PRC2 opposes Merkel cell formation, it is perhaps surprising that the PRC2 catalytic subunit EZH2 has been shown to be expressed in some MCC tumors (205–208), in which context it has been correlated with worse prognosis (206, 207). Activating EZH2 mutations, however, are rare in MCC (10, 42, 43, 86). EZH2 expression can be promoted by E2F transcription factors (209), suggesting a mechanism by which this gene might be upregulated in Rb-deficient tumors such as MCC. Thus far, there is conflicting data on the potential efficacy and mechanism of EZH2 inhibitors in MCC, with 2 reports finding that inhibition of the EZH2 methyltransferase activity had cytotoxic effects on VP-MCC cells and/or xenografts (208, 210), whereas another study found that protein degraders of EZH2 impaired VP-MCC cell line viability independently of the methyltransferase function (211). The antitumor effect of EZH2 inhibition on MCC might be mediated by reversing EZH2-mediated repression of inner ear differentiation genes including *SIX1* (210). Further investigations are needed to better clarify the complex roles for PRC2 and the EZH2 subunit in MCC tumor biology.

The Myc oncoprotein exerts broad effects on transcription patterns in tumors via multiple mechanisms, including epigenetic modification. In keeping with a role for Myc family dysregulation in MCC, *MYCL* (and to a lesser extent *MYC*) can be amplified in a subset of MCC tumors (10, 212). More recently, evidence has shown that MCPyV small T antigen also acts in complex with MYCL and EP400 to promote LSD1 expression. In turn, LSD1 regulates methylation of H3K4 and H3K9 (associated with transcriptional activation and repression, respectively), resulting in gene expression changes that promote tumor proliferation and survival (150, 213). Importantly, Myc overexpression in MCC has been linked to the activating epigenetic mark H3K27Ac in the *MYC* promoter region (214). This represents a potential therapeutic vulnerability, as the H3K27Ac is bound by BRD4, which can be targeted by BET inhibitors to result in antitumor effects in MCC (214–216); however, the Myc-dependency of this effect has been debated (215).

Other histone methylation marks that may be altered in MCC include H3K9 methylation via PRDM8 (217), and induction of histone changes by sTAg (including H3K4Me2, H4K20me2, and the DNA damage associated phosphorylation of H2AX) (218).

Alongside DNA methylation, histone modifications also appear to be an epigenetic mechanism for immune evasion by MCC. Silencing of antigen-presenting machinery in MCC via histone deacetylation results in loss of surface HLA expression, promoting immune evasion (219). Thus, HDAC inhibitors can restore HLA expression and promote immunogenicity of MCC (189, 219–221). H3K9 deacetylation has been associated with silencing of MHC class I chain-related protein, thus removing an activating signal for NK cell targeting of tumor cells (222). Histone methylation may also play a role in HLA silencing in MCC. Specifically, HLA silencing correlates with loss of global H3K27Me3 in MCC, both in fully developed tumors (208, 223), and during the transition of precursor squamous cells into MCC (173); this silencing can be reversed by EZH2 inhibition (223).

Of small regulatory RNAs, evidence thus far supports roles for microRNAs in MCC tumor biology (224). MCCs display distinct patterns of microRNA expression relative to normal tissues and other skin tumor types (225). In addition, microRNA expression patterns may differ between VP-MCC and VN-MCC (226, 227), possibly due in part to microRNA regulation by MCPyV T-antigen(s) (228). A highly expressed microRNA in a subset of MCC is miR-375 (226, 229). Studies have found that miR-375 may contribute to tumor biology in MCC via intracellular and intercellular mechanisms, although reports are mixed regarding the significance and mechanism of this effect (123, 228, 230–234). In addition to these cellular microRNAs, MCPyV encodes a microRNA, MCV-mir-M1-5p, that may suppress LTAg expression and contribute to viral latency and episomal persistence (134, 228, 235, 236).

### 3.6 Cell of origin of MCC

**Figure 2:**
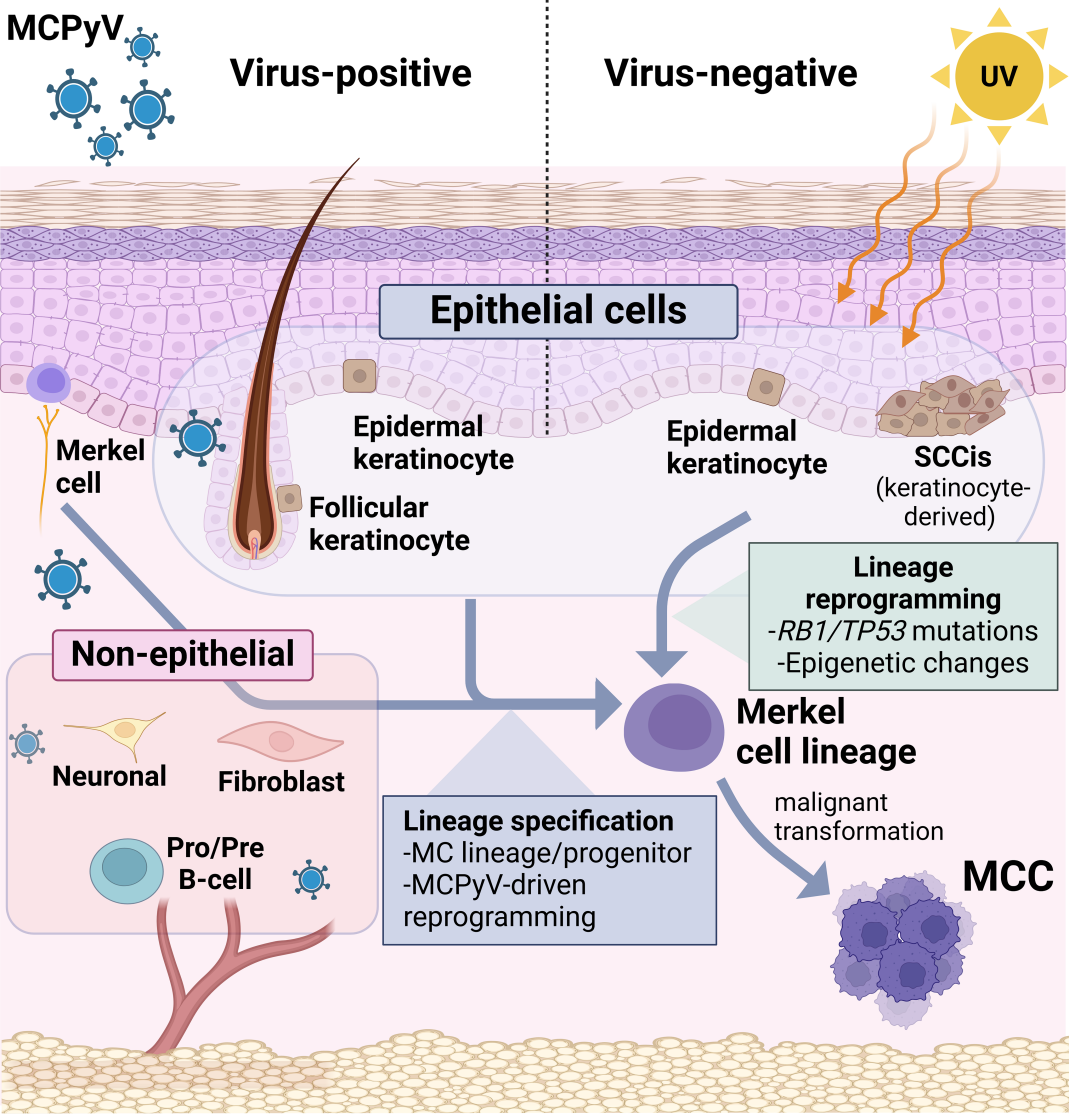
Candidates for MCC cell of origin. (Left) Proposed candidates for VP-MCC include many diverse cell types, especially UV-shielded dermal populations such as follicular epithelial cells and precursors, and non-epithelial lineages including neuronal, fibroblast, and pre-pro B-cells. By contrast, direct observations from human tumors strongly suggest epidermal keratinocytes to be the precursors for VN-MCC. In either scenario, lineage reprogramming (with an as-yet unknown trigger) would be required for a shift to the Merkel cell phenotype in addition to malignant transformation.

MCC was originally named “trabecular carcinoma”, but further investigation soon revealed a high degree of similarity with normal Merkel cells (MCs), prompting a name change to Merkel cell carcinoma (237, 238), and also suggesting these tumors were derived from their namesake. However, multiple lines of evidence now suggest that MCs are not the cell of origin for MCC, including tissue localization, post-mitotic and terminal differentiation status of MCs, differential expression of multiple markers including keratins, as well as the failure of mouse modeling using MC specific drivers (239). Considerable debate about the MCC cell of origin persists, with proposed cells of origin including epithelial, dermal, and lymphoid cell populations (**Figure 2**) (34, 240, 241).

Furthermore, current genomic data suggests VP- and VN-MCC arise from distinct progenitor populations since VN tumors carry a high UV-signature mutational burden (43, 45, 102), which is characteristic of tumors originating in the epidermis. In contrast, VP tumors harbor few UV-mutations, suggesting they are not derived from sun-damaged epidermal cells, and possibly reside at deeper locations (**Figure 2**). This is in keeping with the nascent tumors arising in mice with epidermal-driven expression of TAgs and ATOH1, which appeared spatially restricted to the outer root sheath of the hair follicle, near the bulge compartment (242). The lack of UV-mutations, and the dermal location characteristic of MCC, could also be explained by a non-epithelial cell of origin. Interestingly, dermal fibroblasts have a similar mutational burden as VP-MCCs (239, 243) and thus far are the only host cell that has been shown to support the MCPyV viral life cycle by *in vitro* assays (241). Another non-epithelial candidate cell type with dermal localization is the B-cell lineage, proposed mainly due to MCC expression of early B-cell lineage markers and immunoglobulins (34, 244). Neuronal cells are another possible candidate, since TAg knockdown in VP-MCC cells converts them to a differentiated neuronal phenotype (124).

Although lymphoid or mesenchymal populations have been proposed for the VP-MCC cell of origin, currently, most mouse modeling studies support epithelial populations for this role, since MCPyV TAgs were shown to drive transformation or tumor development in mice from epidermal drivers (117, 118, 138, 242). While murine MCCs only developed upon exogenous ATOH1 expression driving an epidermal-neuroendocrine fate switch, this lineage was also maintained following transient ATOH1 activation (118). This suggests that MCCs may therefore arise from epithelial cells with transitory ATOH1 expression if a neuroendocrine cell fate feed forward loop is established (200, 245, 246). These findings support the concept that commitment to the MC lineage may be a fundamental characteristic of the MCC cell of origin.

Recent data showing mutational overlap between a trichoblastoma and a VP-MCC in a rare combined tumor implies that viral integration in a follicular epithelial cell triggered MCC development (11). Although rare, this case demonstrates the potential for follicular epithelial cells to give rise to human VP-MCC tumors. This aligns with reports of MCPyV protein expression in human follicular epithelial cells, consistent with infection of that cell population by wild-type MCPyV in human skin (247). Furthermore, the appearance of nascent tumors spatially restricted to the hair follicle outer root sheath, in mice with epidermal-driven expression of TAgs and ATOH1, also supports these cells as a potential cell of origin (242).

VN-MCC can display squamous differentiation, or coexist with invasive squamous cell carcinoma or SCCIS (7, 10). Squamous and neuroendocrine areas of differentiation in combined tumors share mutations and copy number abnormalities (159, 172); however, it is not clear to what extent this similarity reflects squamous metaplasia and/or bidirectional tumor differentiation, rather than evidence for MCC arising within a squamous precursor. Recent studies more specifically comparing SCCIS associated with VN-MCC found that both components share key driver mutations in *TP53* and *RB1* (173, 248, 249) and copy number variations (173, 250). Together, these findings suggest that SCCIS is clonally related to the underlying MCC and thus may be a progenitor lesion. However, these studies did not identify a mutational event responsible for the transition from SCCIS to MCC, rather uncovering a shift associated with epigenetic changes (173, 248).

Comprehensive analysis of DNA-methylation patterns in classical VP- and VN-MCC cell lines suggests an epithelial origin for both MCC subtypes (189, 190). Taken together, these findings provide strong support for an epithelial origin for MCC, and further suggest that VP-MCC and VN-MCC might arise from distinct follicular epithelial and epidermal progenitors, respectively.

## 4 Emerging and investigational therapies for Merkel cell carcinoma

Immune therapy has improved the prognosis of advanced and metastatic MCC, but is not universally effective or tolerated. Thus, there is continued need for additional therapeutic options that are effective either alone or that work in combination with immunotherapy. Furthermore, the potential benefit of adjuvant or neoadjuvant immunotherapy in MCC must be defined. Here, we review the current status of therapeutic strategies that are being studied in the clinical setting, based upon the fundamental observations of MCC tumor biology described above.

### 4.1 Adjuvant immunotherapy

Adjuvant immunotherapy trials are underway to determine the efficacy of immunotherapy to prevent disease relapse in treated patients. The ADMEC-O trial (NCT02196961) is evaluating adjuvant nivolumab in treated stage I-IV MCC patients (251). Interim analysis showed a 9% risk reduction at 1 year and 10% at 2 years (252). The STAMP (Surgically Treated Adjuvant Merkel Cell Carcinoma with Pembrolizumab) trial (NCT03712605) is evaluating pembrolizumab as adjuvant therapy in patients with resected stage I-IIIB MCC versus current standard-of-care observation (253). Adjuvant avelumab is being studied in stage III MCC patients in the ADAM (Adjuvant Avelumab in Merkel) trial (NCT03271372) (251). Clinical trial NCT03798639 is studying the effect of nivolumab and radiation or nivolumab and ipilimumab for patients with resected stage IIIA or IIIB MCC (251).

### 4.2 Neoadjuvant immunotherapy

Neoadjuvant therapy trials are underway for MCC. The Checkmate-358 trial (NCT02488759) evaluated neoadjuvant nivolumab in stage IIA-IV MCC (82). Additional trials are ongoing to evaluate the efficacy of the neoadjuvant approach with immunotherapies including pembrolizumab (NCT05496036), pembrolizumab with lenvatinib (NCT04869137), cemiplimab (NCT04975152), and retifanlimab with cisplatin (NCT05594290) (77). Neoadjuvant intratumoral injection with L19IL2/L19TNF (NCT05329792) and PH-762, an RNAi targeting PD-1 (NCT06014086) is also under evaluation (251).

### 4.3 Combination immunotherapy

Combination therapy with nivolumab and the CTLA-4 inhibitor ipilimumab is under evaluation as first-line therapy and in checkpoint inhibitor-refractory disease. A randomized, open label phase 2 trial (NCT03071406) evaluated the combination of ipilimumab and nivolumab with or without stereotactic body radiation therapy (SBRT) as first line or following PD-1/PD-L1 monotherapy (254). This study found ORR in both the first-line and second-line treatment groups. A retrospective study of 14 patients from the German-based multi-center prospective skin cancer registry ADOREG demonstrated a response to combined ipilimumab and nivolumab in Avelumab-refractory patients, demonstrating a progression-free survival rate of 42.9% at 12 months and 26.8% at 24 months (255). Conversely, a retrospective study of 13 patients refractory of checkpoint inhibitor inhibition found only a limited benefit from treatment with combination of ipilimumab and nivolumab (256). Another retrospective study, of patients who received rescue therapy following unsuccessful anti-PD1 therapy, observed objective responses in 4/13 patients treated with anti-CTLA4 in combination with immunotherapy, although some responses were not durable (257). The Checkmate-358 trial (NCT02488759) evaluated ipilimumab in combination with nivolumab for metastatic MCC (251); the results have not been published.

### 4.4 Other immune-based approaches

Given the high immunogenicity of MCC, continued investigations into immune-based approaches other than PD-1/PD-L1 inhibition have substantial promise for identifying effective new therapies. Therapeutic vaccines are an active avenue of investigation for cancer therapy, including MCC (77). In melanoma, a trial evaluating the role of a vaccine against telomerase in combination with pembrolizumab was shown to have preliminary positive results (258). For MCC, vaccines might potentially target either MCPyV epitopes for VP-MCC, or patient-specific tumor neoantigens for VN-MCC (259). Mouse modeling experiments with DNA vaccines based upon LTAg, sTAg, and VP1 peptides have shown promising results (260–263). A phase I DNA vaccine trial utilizing a modified MCPyV LTAg is underway in MCPyV-positive MCC patients (NCT05422781), with results pending (251).

Talimogene laherparepvec (T-VEC) is an injectable engineered oncolytic virus that has been shown to induce an immunologic anti-tumor response in melanoma (264). The efficacy of T-VEC injection is currently in clinical trials as a single agent in locally advanced MCC (NCT03458117), with or without radiotherapy in metastatic lesions of MCC (NCT02819843), and with nivolumab in refractory disease (NCT02978625) (251).

CD47 is a cell surface ligand that opposes antitumor immunity by preventing immune-mediated phagocytosis. *In vitro* expression of CD47 has been shown to be influenced by the sTAg of MCPyV (147). Anti-CD47 therapy has been investigated in a clinical trial for CTCL which also included MCC (NCT02890368) (147).

Other immune-based therapeutic strategies under investigation for MCC include natural killer cell immunotherapy, stimulating tumor MHC expression (via approaches including interferons or epigenetic modifiers), and adoptive T-cell transfer including chimeric antigen receptor (CAR) T-cells (251, 259).

### 4.5 Epigenetic therapy

Epigenetic modulation can normalize cell cycle and apoptosis regulation, thus facilitating removal of aberrant cells. Histone deacetylases (HDACs) are a class of enzymes that play an important role in gene expression. Abnormal regulation and expression of HDACs has been identified in several malignancies. Domatinostat is a HDAC class I inhibitor that has shown anti-tumor effects and upregulation of MHC class 1 molecules in MCC cells (221). Domatinostat in combination with Avelumab is currently being evaluated in the MERKLIN 2 trial for patients with advanced MCC who have progressed on anti-checkpoint inhibitor therapy (NCT04393753) (251). The Bromodomains and Extra-terminal Domains (BET) family of proteins also play a role in gene expression. Both a BET inhibitor and BET degrader, JQ1, and BETd-246, respectively, have demonstrated *in vitro* anti-tumor effect in MCC cell lines (215, 216). Similarly, the hypomethylating agent decitabine has shown anti-tumor effects in MCC cell lines and xenograft tumors (189). Finally, as noted above, activity of the histone demethylase LSD1 (KDM1A) may represent a therapeutic vulnerability for MCC (150, 213). These promising early results underscore the need for further investigation of epigenetic modifiers as therapeutics for MCC.

### 4.6 Targeted therapies in clinical trials

As the underlying mechanisms of MCC tumor biology are more precisely characterized, distinct therapeutic targets will emerge. These might be useful as single agents, in combination with immunotherapy, or for immunotherapy-refractory disease. There have been many trials of investigational agents in both humans and preclinical mouse models based upon biomarker expression patterns and therapeutic performance in other tumor types, as summarized in **Table 2** (89, 149, 197, 205–208, 210, 211, 223, 259, 265–274, 277–279, 283–285, 288–294, 304).

**Table 2:** Emerging targets in clinical and preclinical studies.

*Target*	Rationale	Evidence	*Refs*
***MDM2/4 inhibitors***	VP-MCC typically harbor wild-type p53 that is functionally inactivated via small T antigen-activation of MDM2. Inhibition of MDM2 and MDM4 removes this suppression of p53.	MDM2/4 inhibitors show reduced growth in mouse MCC models. KRT-232 or Navtemadlin, an oral inhibitor of MDM2, is in clinical trial for immunotherapy-naïve or in combination with Avelumab for checkpoint inhibitor-refractory MCC (NCT03787602).	(149) (265),
***EZH2 inhibitors***	EZH2 is a histone methyltransferase that has been shown to be overexpressed in MCC and can play a role in tumorigenesis by epigenetic gene silencing. However, the loss of H3K27Me3 in VN-MCC raises questions regarding the role of EZH2, and thus the potential efficacy of EZH2 inhibitors, in that subset of MCC.	Tazemetostat shows reduced growth of MCC in xenografts.	(197, 205–208, 211, 223, 259)
***Tyrosine kinase inhibitors (TKIs)***	Due to the role of angiogenesis in tumorigenesis, TKIs targeting vascular endothelial growth factor receptor (VEGFR) and related kinases have been investigated as potential therapies for MCC. The tyrosine kinase c-kit is frequently expressed in MCC. However, MCC lacks highly recurrent tyrosine kinase amplification or mutation events that would otherwise provide a rationale for targeted tyrosine kinase inhibitor (TKI) therapy. molecular studies have not demonstrated a significant rate of activating mutations of the *KIT* gene, the tyrosine kinase c-kit is frequently expressed in MCC.	Apatinib showed clinical benefit in a case report of one patient with recurrent MCC. Imatinib provided benefit in two case reports. Pazopanib showing temporary benefit in a case report of a patient with MCC. 5 of 24 patients with metastatic MCC demonstrated a clinical response to either pazopanib or cabozantinib (case series). A clinical trial of the multi-kinase inhibitor cabozantinib in advanced MCC (NCT02036476) was halted to due toxicities and lack of response. A recent trial including kinase inhibitors as salvage therapy in patients who failed first-line immunotherapy failed to show response. In a phase II trial, patients showed progression of disease on imatinib.	(266–276)
***Anti-apoptosis inhibitors***	Pro-survival molecules including Bcl-2 family of proteins and inhibitor of apoptosis proteins (IAP) contributes to tumor biology by prevention of apoptosis. It has been shown that inhibition of these proteins facilitate apoptosis in MCC cells.	Navitoclax, a Bcl-2 family inhibitor showed *in vitro* efficacy in combination with a PI3K kinase inhibitor. Alpelisib induces apoptosis *in vitro* and in mouse models. The novel bcl-2 antisense agent (G3139, Genasense) showed decreased growth in a mouse model. However, a phase II trial of Genasense failed to show efficacy.	(277–282)
***Epigenetic modifiers***	Histone deacetylase inhibition, histone demethylase inhibition, and hypomethylating agents are an active area of development for many cancers. MCC harbors numerous epigenetic changes as detailed in the text. The sTAg activates the lysine-specific histone demethylase (LSD1) and is required for tumorigenesis.	Multiple LSD1 inhibitors inhibit MCC growth *in vitro* and in xenograft models. The hypomethylating agent decitabine inhibited virus-positive MCC cells *in vitro* and in xenograft models. Histone deacetylase inhibition combined with PD-1/PD-L1 blockade did not show clinical benefit in a series of four patients.	(189, 213, 219, 222)
***Antibody-drug conjugates***	Antibody-drug conjugates are emerging as a method of treatment for malignancies, including MCC. CD56 is a glycoprotein expressed on neuroendocrine tumors, and has been shown to be expressed on MCC tumors. DLL3 has also been proposed as a target for antibody-drug conjugates.	A conjugate that combines a CD56-targeting antibody to the cytotoxic drug, monomethyl auristatin E inhibits growth in mouse xenograft models of MCC. A Phase I clinical trial using a different (subsequently discontinued) CD56-targeting conjugate reported responses in a minority of patients with MCC.	(283–287)
***Somatostatin analogs***	Somatostatin analogs have demonstrated efficacy in the treatment of neuroendocrine tumors. Somatostatin receptors are expressed in MCC. Somatostatin analogs are an active area of investigation, especially those paired with radionuclides.	Somatostatin analogs have shown clinical benefit in some MCC patients, although the rates of response to somatostatin analogs as monotherapy has been low.	(288–290)
***JAK inhibitors***	Janus kinases (JAK) are the intracellular non-receptor tyrosine kinases of the JAK-STAT pathway. A World Health Organization (WHO) international database query identified a disproportionate increase in MCC diagnoses among patients taking the JAK inhibitors ruxolitinib, tofactinib, and baricitinib.	Case reports have validated increased MCC diagnoses in patients treated with JAK inhibitors. However, case reports have also described a robust tumor response upon addition of nivolumab despite continuation of ruxolitinib. Thus, it is currently unclear whether ruxolitinib is antagonistic or synergistic to anti-PD1 therapy.	(291–294)
***Ras inhibitors***	Ras signaling downstream of receptor tyrosine kinase activity may be amenable to treatment.	Farnesylthiosalicylic acid was associated with reduced growth in MCC xenografts.	(295)
***Bromodomain inhibitors***	BET family members Brd2, Brd3, and Brd4 are expressed in MCC and upregulate MYC. BET protein inhibitors are being actively developed for several cancers	JQ1 inhibits BET proteins and blocked MCC xenograft growth.	(214–216, 296)
***HSP70 inhibitors***	LTAgs require heat shock protein 70 (HSP70) family members to exert their transforming activity.	The HSP70 modulator MAL3-101 inhibits tumor growth *in vitro* and in MCC xenograft models.	(297)
***PI3K/Akt/mTOR inhibitors***	Activation of the phosphatidylinositide-3-kinase (PI3K)/AKT/mTOR pathway is frequently detected in MCC	MLN0128 suppress mTOR signaling and blocked MCC xenograft growth. Copanlisib inhibits PI3K signaling *in vitro* and in a MCC xenograft model.	(157, 162, 296, 298)
***ABCB5 inhibition***	ATP–binding cassette member B5 (ABCB5) mediates chemoresistance. ABCB5 expression was detected in MCC cell lines and tumors at levels significantly higher than those in normal skin. Carboplatin- and etoposide-resistant MCC cell lines exhibited increased expression of ABCB5.	ABCB5 blockade inhibits growth in MCC xenograft models.	(299)
***Serine/ threonine kinase inhibitors***	Aurora kinase A is being investigated as a target in many cancers. Glycogen synthase kinase (GSK3) may be required for viral T antigen function.	AK-01/LY3295668 (Aurora kinase A inhibitor) and CHIR99021 (GSK3 inhibitor) block MCC growth *in vitro* and in xenograft models.	(300, 301)
***MUC1-C inhibitor***	MUC1-C is broadly expressed in MCCs and regulates common sets of signaling pathways related to RNA synthesis, processing, and transport.	GO-203 inhibits MUC1-C and blocks tumor growth *in vitro* and in MCC xenograft model.	(302)
***Pyrvinium pamoate***	The FDA-approved antihelminthic drug that was identified in a computational drug screen. It was shown to inhibit Wnt signaling, activate p53-mediated apoptosis, and disrupt mitochondrial function.	Pyrvinium pamoate inhibited tumor growth in a MCC xenograft model.	(303)

## 5 Preclinical mouse models of MCC

**Figure 3:**
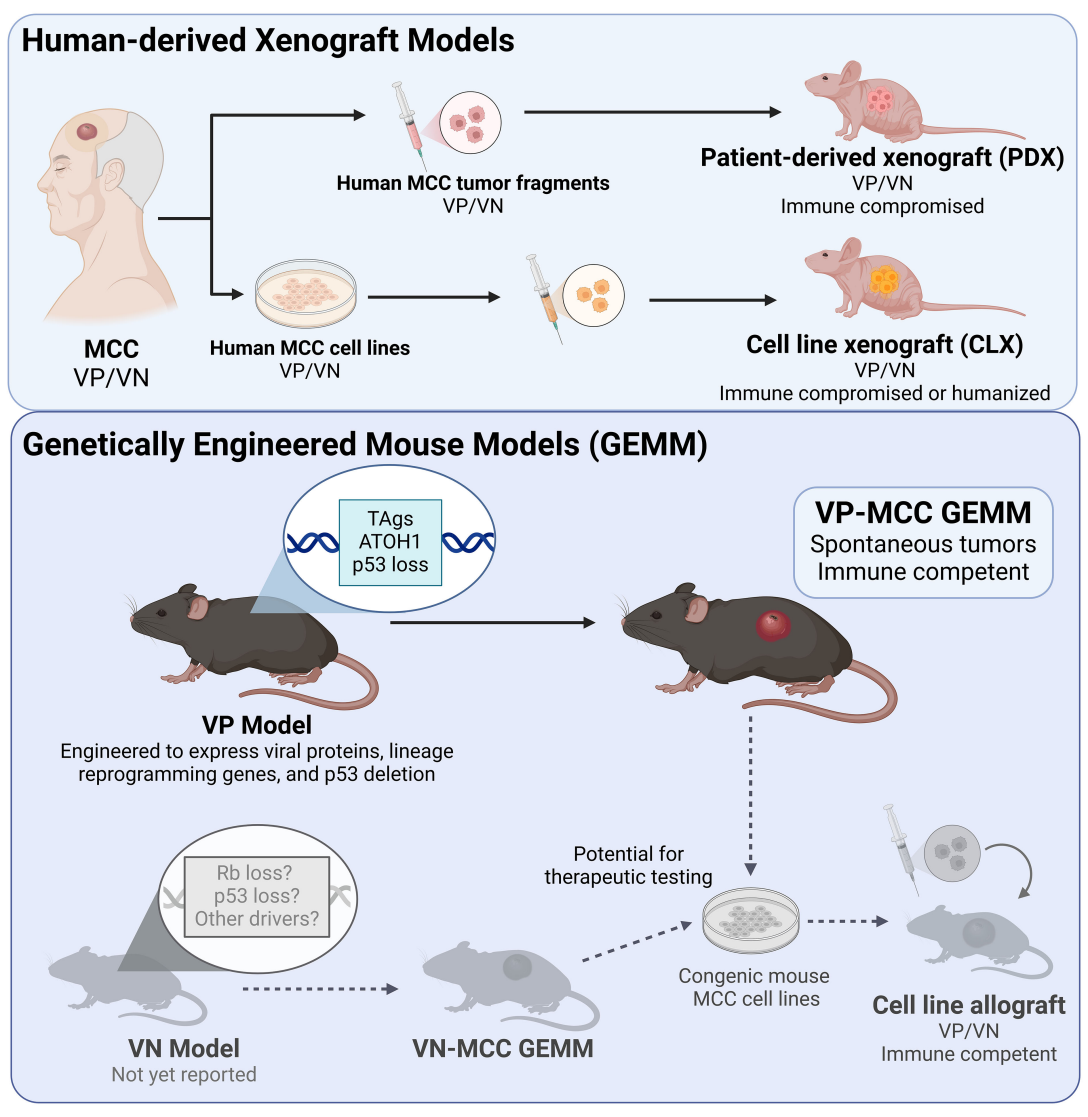
Mouse models of MCC. (Top panel) Mouse models of MCC utilizing xenografts of human MCC include patient-derived xenograft models in immune compromised mice, and cell line xenografts in immune compromised or humanized mice. Of these, only humanized mice allow for assessment of antitumor immune response. (Bottom panel) Generation of a murine virus positive (VP) MCC tumor required genetically engineered mice expressing MCC oncoproteins (TAgs) accompanied by ATOH1 for neuroendocrine reprogramming, and inactivation of p53. The resulting tumors can be studied in immune competent mice, allowing for studies of antitumor immunity (such as for immunotherapy). There is currently no virus negative (VN) MCC mouse model, however such a model would presumably require inactivation of the *Trp53* and *RB1* tumor suppressor genes similar to human VN-MCC tumors. Generation of congenic mouse cell lines from VP and VN GEMMs would allow pre-clinical therapeutic studies in an immune competent syngeneic model. Examples in gray have not yet been reported.

Accurate and reliable preclinical models are invaluable for testing new therapeutic strategies prior to testing in humans. While a better understanding of the molecular pathogenesis of MCC has led to improve *in vitro* and *in vivo* models since the discovery of the MCPyV [reviewed in (305)], multiple questions remain that could be addressed by mouse models. Types of murine models used in cancer research include cell line xenografts (CLX), patient-derived xenografts (PDX), syngeneic models, and genetically engineered mouse models (GEMMs)(**Figure 3**). Each provides different opportunities or poses specific challenges for investigating tumor initiation, progression, and metastases as well as possible prevention and therapeutic strategies. Successful development of these models is critical to advance understanding of both viral and UV-driven MCC tumors.

### 5.1 Transplantable MCC mouse models

Establishment of cancer cell lines from human tumors has traditionally allowed for the development of CLX models in immunocompromised mice, which have classically been powerful tools for drug efficacy and toxicity studies, including in MCC (150, 189, 213, 215, 216, 219, 222, 265, 275, 277, 280–282, 284, 295–303).

While the rarity of MCC may in part be responsible for the lack of PDX models to date, several recent studies now provide compelling preclinical data for the use of targeted monotherapies or potential combinatorial regimens in select MCC patients. The PI3K/mTOR/AKT pathway is commonly activated in MCC, and use of the PI3K inhibitor copanlisib, specifically targeting PI3K-α and PI3K-δ isoforms, showed antitumor effects in multiple MCC PDX models compared to other PI3K inhibitors (298). CDK4/6 inhibition with palbociclib increases PD-L1 expression, and when combined with HIF2alpha inhibition results in growth inhibition using both CDX models and a PDX-derived cell line (306). Additionally, PDX models have proven valuable for assessing MDM2 inhibition via the small molecule inhibitor, milademetan, in MCCs with wild-type p53 (265).

Though classically used for therapeutic strategizing, CLX and PDX models have additionally provided opportunities for establishing and characterizing MCC cell lines (277, 307–309). They have been valuable for observing the appearance of circulating MCC tumor cells and metastasis formation (310), determining the requirement of TAg expression in MCC cells (136), as well as for studying vaccine development (260, 261), vasculogenic mimicry as a novel MCC biomarker (311), and circulating microRNA miR-375 as a surrogate biomarker (230). Xenograft models have also helped establish the role of cancer-associated fibroblasts in promoting MCC progression (312).

### 5.2 Genetically engineered mouse models of MCPyV tumorigenesis

Undoubtedly, transplantable mouse models can be powerful in specific settings. However, a GEMM that develops spontaneous MCC tumors recapitulating the human cancer will provide the best insight into tumor development and reveal possibilities for prevention and treatment. The clonal integration of MCPyV sequences expressing TAgs in MCC supports this virus as the driver of tumorigenesis. However, despite multiple attempts to capitalize on MCPyV TAg expression in mice over the years (117, 118, 138, 139, 242, 313, 314), generation of a faithful, accessible MCC GEMM has been complicated and hindered by the unknown cell of origin.

The *in vitro* transforming potential of sTAg (135) was subsequently corroborated in transgenic mice expressing sTAg (117). Although this study did not yield MCC tumors, it was the first evidence in a mouse model that sTAg expression is sufficient for epithelial transformation. Using a Keratin 5 (K5) promoter to drive expression of wild-type or mutant sTAgs in epidermis, this model revealed that the transformation phenotype was dependent on the sTAg LSD-binding domain. These sTAg transforming studies, initially performed in a preterm embryo model, were validated in an adult model that rapidly developed epidermal hyperplasia and advanced skin lesions with histological changes that collectively recapitulated human squamous cell carcinoma *in situ* (SCCIS), also known as Bowen’s disease (117).

That same year, two additional studies implicated the TAgs in cancer formation (138, 139). Use of an epithelial tissue-specific conditional mouse model further demonstrated that a K14-driven MCPyV early region expressing TAgs has tumorigenic activity *in vivo*. These mice also developed epidermal hyperplasia, with some advancing to cutaneous papillomas (138). Another mouse model generated to conditionally express sTAg from the ROSA26 locus revealed ubiquitous sTAg was lethal at high levels, while lower expression could induce a hyperplastic skin phenotype (139). Expression of ubiquitous sTAg in the context of a homozygous p53 deletion in this model generated poorly differentiated malignancies in spleen and liver, however, MCC development was not observed.

Taken together, these initial mouse models demonstrated the capability of sTAg to drive undifferentiated malignancy, epidermal hyperplasia, and epidermal dysplasia, but did not culminate in a neuroendocrine tumor. The lack of MCC development suggests the TAgs might not have been targeted to the appropriate cell population or required the contribution of additional factors. An early theory of MCC origin (no longer considered likely) posited that these represent malignant transformation of Merkel cells (MCs), due to their shared structural and immunohistochemical characteristics (239). However, sTAg-targeted to embryonic or adult Merkel cells showed no sustained proliferative capacity and no transforming potential, even in the p53 null setting (139, 239). This is consistent with most MCs being post-mitotic or terminally differentiated, and thus unlikely to generate a neoplastic proliferation (315).

Merkel cells themselves have been shown to be derived from an epidermal progenitor in an ATOH1 dependent manner (316, 317), and epidermal expression of ATOH1 in embryonic and adult mice drives formation of ectopic Merkel cells (245). MCC tumors express ATOH1 (27, 28, 30), suggesting that these tumors might arise from epidermal progenitors in a process that recapitulates the differentiation pathway of normal Merkel cells. This insight led to a key advance in MCC mouse modeling. When keratinocytes were reprogrammed to the MC lineage by ATOH1, sTAg expression was sufficient to initiate small blue cell tumors resembling human intraepidermal MCC in late-stage mouse embryos (118). These MCC-like tumors expressed MC markers and K8 and K20 in a clumped or dot-like pattern typically detected in MCC tumor cells (7, 318).

This finding was followed up by a study designed to ascertain whether TAg-expressing epidermal cells reprogrammed to the MC lineage via ATOH1 would develop murine MCC tumors in adult mice (242). These conditional mice expressing sTAg, tLTAg and ATOH1 in *Krt5*-expressing cells and their progeny, yielded microscopic collections of proliferating MCC-like cells, but no gross tumors.

An important modification of this mouse model was additional targeted deletion of *Trp53* (**Figure 3**). This change, which ensured the loss of p53 activity known to be critical in human MCC tumors, was the final step to yield macroscopic skin tumors with classic MCC histology including a monomorphous small blue cell phenotype, finely stippled chromatin, prominent mitoses, and nuclear molding. Furthermore, they mimicked human MCC tumors at the marker expression level, and cross-species transcriptomic analysis revealed close similarity to human tumors. Thus, this model which relies on epidermal expression of MCPyV TAgs, ectopic expression of ATOH1 to drive epidermal reprogramming, and loss of p53, is the first GEMM to successfully develop murine MCC (242).

### 5.3 Unmet needs in translational mouse models

Despite the initial promise raised by CLX and PDX studies, targeted approaches for MCC have performed poorly in clinical trials (267, 270, 274, 279, 289), and there is currently no approved targeted therapy for advanced MCC. Since CLX models do not necessarily accurately recapitulate the phenotypic and molecular heterogeneity of MCC tumors, use of PDX models utilizing patient tumor material may be more representative and better predictors of clinical responses to anticancer agents (319–321). Importantly, both CLX and PDX models require immune depleted mice, thus they are not informative for immunotherapy strategies. Preclinical immunotherapy studies are best tested in a mouse model where an intact immune system is present.

For these reasons, “humanized” mouse models, consisting of immune deficient mice with various human immune cell engraftments, used in conjunction with CLXs or PDXs have become fundamentally important in onco-immunology studies (322, 323). Use of a humanized mouse model has suggested the potential application for T-cell-mediated immunotherapy directed against MCPyV TAgs (324). Although not yet extensively used for MCC studies, such humanized mice may provide a powerful platform for testing responsiveness to novel immunotherapies or combination regimens, as well as better predicting clinical responses.

Another alternative strategy is to develop syngeneic models utilizing immune-competent animals. Syngeneic mouse models utilize immune-competent mice that develop tumors derived from murine cancer cells that have been engrafted from genetically identical congenic mouse strains. Syngeneic models are typically considered the workhorse for investigating immunotherapies and studying the immune surveillance of cancer development (322, 325, 326). Since the only current FDA-approved MCC therapies target the PD-1/PD-L1 (programmed cell death protein 1/PD1 ligand) immune checkpoint pathway, which benefits ~50% of MCC patients (73, 75, 76, 78, 327), a syngeneic model would be a tremendous benefit and provide a much-needed immunotherapy pre-clinical model. However, currently there are no syngeneic MCC mouse models available.

To date, MCC viral-driven mouse modeling has focused on expression of individual or TAg combinations, however the role of each has not been fully dissected. sTAg in the absence of LTAg was shown to be the primary oncogenic driver in several mouse models (117, 118, 139). The precise contribution of tLTAg in the latest murine MCC model (242) is currently unknown. A recent study utilizing the previously developed *K14*-driven transgenic mouse expressing both sTAg and tLTAg (138) indicates the resulting epithelial phenotypes are dependent upon the LTAg-pRb interaction (313). The functional role of TAgs was additionally investigated in a multi-stage chemically-induced mouse model of skin carcinogenesis, revealing that expression of TAgs in stratified epithelia synergize with the tumor-inducing agent dimethylbenz(a)anthracene (DMBA), but not the tumor promoter 12-*O*-tetradecanoylphorbol-13-acetate (TPA), suggesting TAgs operate predominantly as tumor promoters (314). Exactly how the MCPyV TAgs contribute to MCC tumorigenesis remains an open question, and poorly understood or disparate functions of TAgs *in vivo* may be a consequence of experimental design and modeling strategies which do not truly recapitulate human MCCs.

The unexpected requirement for the loss of p53 to drive full blown virus-positive murine tumors remains perplexing (242). While *TP53* mutations are common in UV-driven MCCs, they are rare in viral-driven MCC (10, 43, 45, 102) conceivably because sTAg upregulates MDM2 and the MDM4 activator CK1α to repress p53, thus abrogating the need for mutational inactivation (149). Interestingly, the requirement for the loss of p53 in sTAg-induced transformation was seen previously in mouse embryonic fibroblasts and in mice, and it was hypothesized that sTAg induces mitotic catastrophe and p53 activation leading to apoptosis (139). The presence of activated p53 and apoptotic cells in microscopic lesions of adult mice expressing sTAg, tLTAg and ATOH1 potentially supports this theory (242). However, the differential requirement for the loss of *Trp53* in human and mouse TAg-driven tumors may be a result of divergence in p53-regulated targets between the species (328). Regardless of mechanism, abrogating p53 activity in viral-driven MCC appears to be critical.

The development of murine MCCs established a fundamental role for the TAgs in viral-driven MCC tumorigenesis. However this model is dependent on the shift of TAg-expressing epidermal cells into a Merkel cell fate via ectopic ATOH1 expression (242). While this highlights the novelty of manipulating cell fate to generate tumors of unknown origin, it does not accurately reflect MCC development in humans. Although expression of MCPyV TAgs in Sox9-positive epidermal cells has recently been shown to drive hyperproliferative lesions and reprogram cells to express neuroendocrine markers, full blown MCC tumors were not observed (125). Determining whether other types of skin cells can be ATOH1-reprogrammed to yield MCCs, or whether different candidate cells of origin are competent to form MCCs without exogenous ATOH1 or p53 deficiency, remains an active area of research.

Effectively modeling VN-MCC in mice remains challenging. Although transplantable VN-MCC mouse models have been successfully generated and utilized (277), there are currently no GEMMs reported in the peer-reviewed literature for this tumor subtype. While the frequent inactivation of *TP53* and *RB1* genes in human VN-MCC tumors indicates this will likely be a prerequisite for development of a VN-MCC GEMM, additional drivers or pathway perturbations that might be required are unclear. Furthermore, the unknown cell of origin and speculation, that VP- and VN-MCCs arise from distinct progenitor populations (2, 173, 239, 248), suggest this mouse model may be particularly challenging to develop.

## 6 Summary/conclusion

The pathogenesis of MCC has remained poorly understood. Recent studies have fundamentally advanced our understanding regarding potential cells of origin for MCC, the role of epigenetic modifications in tumor biology, and potential therapeutic options. Remaining challenges include more conclusive evidence for MCC origins, mouse models for VN-MCC, syngeneic mouse models suitable for immunotherapy research, and effective therapies for patients who fail to respond to, or tolerate, immunotherapy.

## Author contributions

EP: Conceptualization, Supervision, Visualization, Writing – original draft, Writing – review & editing. MV: Conceptualization, Data curation, Methodology, Project administration, Visualization, Writing – original draft, Writing – review & editing. MJ: Writing – original draft, Writing – review & editing. KH: Writing – original draft, Writing – review & editing. PH: Conceptualization, Project administration, Resources, Supervision, Visualization, Writing – original draft, Writing – review & editing.
